# Tumor Content Chart-Assisted *HER2/*CEP17 Digital PCR Analysis of Gastric Cancer Biopsy Specimens

**DOI:** 10.1371/journal.pone.0154430

**Published:** 2016-04-27

**Authors:** Keisuke Matsusaka, Shumpei Ishikawa, Atsuhito Nakayama, Tetsuo Ushiku, Aiko Nishimoto, Masayuki Urabe, Nobuyuki Kaneko, Akiko Kunita, Atsushi Kaneda, Hiroyuki Aburatani, Mitsuhiro Fujishiro, Yasuyuki Seto, Masashi Fukayama

**Affiliations:** 1 Division of Diagnostic Pathology, the University of Tokyo Hospital, Tokyo, Japan; 2 Department of Molecular Oncology, Graduate School of Medicine, Chiba University, Chiba, Japan; 3 Department of Pathology, Graduate School of Medicine, the University of Tokyo, Tokyo, Japan; 4 Department of Genomic Pathology, Medical Research Institute Tokyo Medical and Dental University, Tokyo, Japan; 5 Department of Gastrointestinal Surgery, the University of Tokyo Hospital, Tokyo, Japan; 6 Genome Science Division, Research Center for Advanced Science and Technology, the University of Tokyo, Tokyo, Japan; 7 Department of Endoscopy and Endoscopic Surgery, the University of Tokyo Hospital, Tokyo, Japan; INRS, CANADA

## Abstract

Evaluating *HER2* gene amplification is an essential component of therapeutic decision-making for advanced or metastatic gastric cancer. A simple method that is applicable to small, formalin-fixed, paraffin-embedded biopsy specimens is desirable as an adjunct to or as a substitute for currently used HER2 immunohistochemistry and in situ hybridization protocols. In this study, we developed a microfluidics-based digital PCR method for determining *HER2* and chromosome 17 centromere (CEP17) copy numbers and estimating tumor content ratio (TCR). The *HER2*/CEP17 ratio is determined by three variables—TCR and absolute copy numbers of *HER2* and CEP17—by examining tumor cells; only the ratio of the latter two can be obtained by digital PCR using the whole specimen without purifying tumor cells. TCR was determined by semi-automatic image analysis. We developed a Tumor Content chart, which is a plane of rectangular coordinates consisting of *HER2*/CEP17 digital PCR data and TCR that delineates amplified, non-amplified, and equivocal areas. By applying this method, 44 clinical gastric cancer biopsy samples were classified as amplified (n = 13), non-amplified (n = 25), or equivocal (n = 6). By comparison, 11 samples were positive, 11 were negative, and 22 were equivocally immunohistochemistry. Thus, our novel method reduced the number of equivocal samples from 22 to 6, thereby obviating the need for confirmation by fluorescence or dual-probe in situ hybridization to < 30% of cases. Tumor content chart-assisted digital PCR analysis is also applicable to multiple sites in surgically resected tissues. These results indicate that this analysis is a useful alternative to HER2 immunohistochemistry in gastric cancers that can serve as a basis for the automated evaluation of *HER2* status.

## Introduction

Gastric cancer is one of the most common malignant tumors and the third-leading cause of cancer-related death throughout the world [1]. Advanced cases without operative intervention have poor prognosis and necessitate multidisciplinary treatment approaches. HER2 is a 185-kDa transmembrane glycoprotein that is a member of the epidermal growth factor receptor family and functions as a tyrosine kinase receptor [2, 3]. In normal cells, HER2 protein acts as a signal transducer during cell proliferation or differentiation [4]; however, it has oncogenic properties when overexpressed. This is usually caused by *HER2* gene amplification, which has been reported in several types of malignant tumor [5] including breast cancer [6, 7], salivary gland adenocarcinoma [8], urinary bladder cancer [9], and gastric cancer [10]. HER2-overexpressing gastric cancers account for 8%–31% of all cases [11–15]. In the Trastuzumab for Gastric Cancer trial, trastuzumab (Roche Diagnostics, Basel, Switzerland)—a humanized anti-HER2 monoclonal antibody—increased the survival rate of patients with HER2-positive gastric cancers when administered in combination with chemotherapy [16], and was also approved for the treatment of breast cancers [17, 18]. Evaluating HER2 status is therefore an essential aspect of gastric cancer therapy.

For patients with unresectable gastric cancers, HER2 status should be examined using a small biopsy specimen. As represented in the latest recommendations for HER2 testing in breast cancer by American Society of Clinical Oncology (ASCO)/College of American Pathologists (CAP), immunohistochemistry (IHC) and/or in situ hybridization (ISH) are the standard methods for evaluating HER2 status [19], but each has its limitations. For instance, the effectiveness of HER2-IHC is affected by several factors such as the formalin fixation process, and the scoring system is not always reproducible especially in equivocal cases, for which ISH is recommended [19–25]. In bright field *HER2* dual-probe ISH (*HER2*-DISH), *HER2* and chromosome 17 centromere (CEP17) copy numbers can be detected as signals under a light microscope over the entire region of the specimen. However, although this method has superior sensitivity, it is costly and relatively time-consuming.

In this study, we applied microfluidics-based digital PCR technology (BioMark; Fluidigm, Cambridge, UK) [26] to determine *HER2*/CEP17 copy number ratio. In digital PCR, template DNA is divided into 770 pieces in nanoliter reaction chambers, with an expected concentration of 0–1 molecule per chamber. Dual-colored target and reference genes are simultaneously PCR-amplified and their copy numbers are calculated by counting the chambers with positive signals. This highly robust method enabled the comparison of *HER2* and CEP17 copy numbers using different primer sets. The objective of this study was to confirm the feasibility of digital PCR technology for analysis of gastric cancer biopsy specimens. However, one problem with this approach is that the tissue usually contains non-cancerous stromal cells that interfere with molecular analyses. To overcome this issue, we developed two-dimensional scatter plot referred to as the Tumor Content (TC) chart that allocates data into amplified, non-amplified, and equivocal categories. The objective was to develop an automated means of evaluating *HER2* status, with TC chart-assisted *HER2*/CEP17 digital PCR analysis serving as the first step.

## Materials and Methods

### Ethics Statement

This study was approved by the University of Tokyo Institutional Ethical Committee. Clinical samples with written informed consent were collected under the University of Tokyo Institutional guidelines for the study of human tissues.

### Clinical gastric cancer biopsy samples and surgical specimens

In total, 44 gastric cancer biopsy specimens from 32 patients processed by routine formalin-fixed paraffin-embedded (FFPE) were available for analysis. All biopsy specimens were obtained endoscopically at the Department of Endoscopy and Endoscopic Surgery. Four surgical specimens, which were resected surgically at the Department of Gastrointestinal Surgery, were also analyzed. These specimens were fixed in 10% neutral buffered formalin. All cases were diagnosed at the Department of Pathology of the University of Tokyo Hospital. Sections were cut at a thickness of 4 μm by using conventional histological techniques; these were collected on glass slides and used for hematoxylin-eosin (HE) staining, HER2-IHC, and *HER2*-DISH.

### Immunohistochemistry

Immunohistochemical staining was carried out on FFPE tissue using Ventana BenchMark XT (Roche Diagnostics) with the 4D5 anti-HER2 antibody. Staining intensity and membrane immunoreactivity patterns were evaluated using the Dako scoring system according to ASCO/CAP guidelines [19]. No membrane staining was scored as 0; faint or barely perceptible/incomplete membrane staining was scored as 1+; incomplete and/or weak/moderate circumferential membrane staining was scored as 2+; and completely and intensely circumferential membrane staining was scored as 3+.

### *HER2-*DISH staining and evaluation

*HER2*-DISH was performed on FFPE tissue by using the HER2 DNA DISH kit (Roche Diagnostics) according to the manufacturer’s instructions, and specimens were evaluated by light microscopy. *HER2* signals appeared as black dots or clusters, and CEP17 signals appeared as red dots. Twenty non-overlapping cancer nuclei were scored for *HER2* and CEP17 signals and *HER2* gene amplification was classified as recommended by the ASCO/CAP guidelines [19]; positive, [*HER*2/CEP17 ratio ≥ 2.0] and/or [average *HER*2 copy number ≥ 6.0 signals/cell]; equivocal, [*HER*2/CEP17 ratio < 2.0] and [average *HER*2 copy number ≥ 4.0 and < 6.0 signals/cell]; negative, [*HER*2/CEP17 ratio < 2.0] and [average *HER*2 copy number < 4.0 signals/cell].

### Preparation of DNA from FFPE clinical material

To extract DNA from biopsy samples, 10 serial sections cut at a thickness of 10 μm were placed on a glass surface for HER2-IHC and *HER2*-DISH. Tumor content was confirmed before and after sectioning by staining the adjacent slides with HE. Since a single paraffin block usually contains several pieces of biopsy sample, individual specimens were isolated using a glass cutter. To extract DNA from surgical resected specimens, 5 serial sections from each representative tissue-block were cut at a thickness of 10 μm, which were placed on a sheet made from polytetrafluoroethylene (PTFE, Sanplatec, Osaka, Japan). The membrane is chemical resistance for xylene-based deparaffinization, and is easily cut into pieces. In the study, various parts were clip out from the sheets according to HER2 profile. Glass or PTFE pieces were assembled in a 2.0-ml tube, and DNA was extracted using the QIAamp DNA FFPE Tissue Kit (Qiagen, Valencia, CA, USA).

### *HER2* copy number assessment by digital PCR

Primer sets for amplifying *HER2* and CEP17 are shown in Table 1. Each primer was designed such that the PCR products obtained were sufficiently short (59 and 65 bp) to eliminate the influence of DNA fragmentation in the FFPE process. Digital PCR was carried out using an EP1 system with a 48.770 digital array integrated fluidic circuit (Fluidigm). Each chip contained 48 panels that were further partitioned into 770 reaction chambers. The number of positive fluorescent signals in each panel was used to quantify different DNA sequences. The protocol has been described elsewhere [26]. Briefly, 4-μl reaction mixtures were prepared for each assay that contained 1× TaqMan gene expression master mix, 1× HER2-FAM and chr17cent-VIC TaqMan probes, and 1× sample loading reagent (Fluidigm). The reaction mixture was evenly distributed into the 770 reaction chambers and the digital array was thermo-cycled on an EP1 System FC1 cycler. The two target regions were independently amplified and 6-carboxyfluorescein and VIC dye signals in the chambers were recorded at the end of each PCR cycle. The number of 6-carboxyfluorescein-positive (HER2) and VIC-positive (CEP17) chambers in each panel was counted and *HER2*/CEP17 copy number ratio was calculated [27].

**Table 1:** Primer and TaqMan probe sequences for digital PCR.

**Gene**	**Primer types**	**Primer sequence**	**Product (bp)**
*HER2*	Fwd	CCCTCCGTACTTCCTGATGCT	59
	Rev	GCCATGGAGAGCCTCACATT	
CEP17	Fwd	CGCTCCTGCACTGTAACACGT	65
	Rev	TCATTCCTGCAGCCCTTGA	
**TaqMan probes**	**Probe sequence**		
*HER2*	TGAGAGTCAAGATCTC		
CEP17	AGCAGGTCCAGCCCA		

### Evaluation of tumor content ratio (TCR)

Whole slide image of HE sections, which were those just before the serial sections for DNA extraction, was scanned using NanoZoomer (Hamamatsu Photonics, Hamamatsu, Japan). Data were imported into Definiens Tissue Studio 2.0 (http://www.tissuestudio.com) for determination of TCR. The selected region of surgical specimens was scanned using a BZ-710 microscope (Keyence, Osaka, Japan). Data were imported into Hybrid Cell Count software (BZ-H3C, Keyence). Data that detailed the number of cells in imported images and distinguished cancerous from non-cancerous cells based on nucleus size were automatically generated

### Experiments for developing the TC charts

#### Cell lines

For control experiments, H522 lung cancer and SK-BR-3 breast cancer cell lines were obtained from the American Type Culture Collection (Manassas, VA, USA). GM18997 Epstein-Barr virus-transformed lymphoblastoid cell line (LCL) was obtained from Coriell Biorepository (https://catalog.coriell.org/). H522 cells and the LCL were cultured in Roswell Park Memorial Institute-1640 medium (Nacalai Tesque, Kyoto, Japan) containing 10% fetal bovine serum (Sigma-Aldrich, St. Louis, MO, USA) and 1% penicillin-streptomycin solution (Nacalai Tesque). SK-BR-3 cells were cultured in Dulbecco’s Modified Eagle’s medium/Hams F12 (Nacalai Tesque, Japan) with 10% fetal bovine serum and 1% penicillin/streptomycin. Cells were cultured at 37°C in an atmosphere containing 5% CO_2_. DNA was extracted using a QIAquick DNA mini kit (Qiagen). FFPE H522, SK-BR-3, and LCL cell blocks were prepared for *HER2*-DISH evaluation.

#### Stepwise mixing with LCL

Genomic DNA from H522 and SK-BR-3 cells and the LCL was adjusted to a concentration of 50 ng/μl. H522 and LCL genomic DNA was mixed in eight steps at the following volume ratios: 8:2, 7:3, 6:4, 5:5, 4:6, 3:7, 2:8, and 1:9 (H522:LCL). SK-BR-3 and LCL genomic DNA was mixed in four steps at the following volume ratios: 8:2, 6:4, 4:6, and 2:8 (SK-BR-3:LCL).

### Statistical analyses

Chi-squared tests were applied to compare counts between *HER2*-DISH and digital PCR in H522 and SK-BR-3 cells and LCL. Differences were considered to be significant if *p* < 0.05.

## Results

### Development of the TC chart for digital PCR data

**Fig 1:**
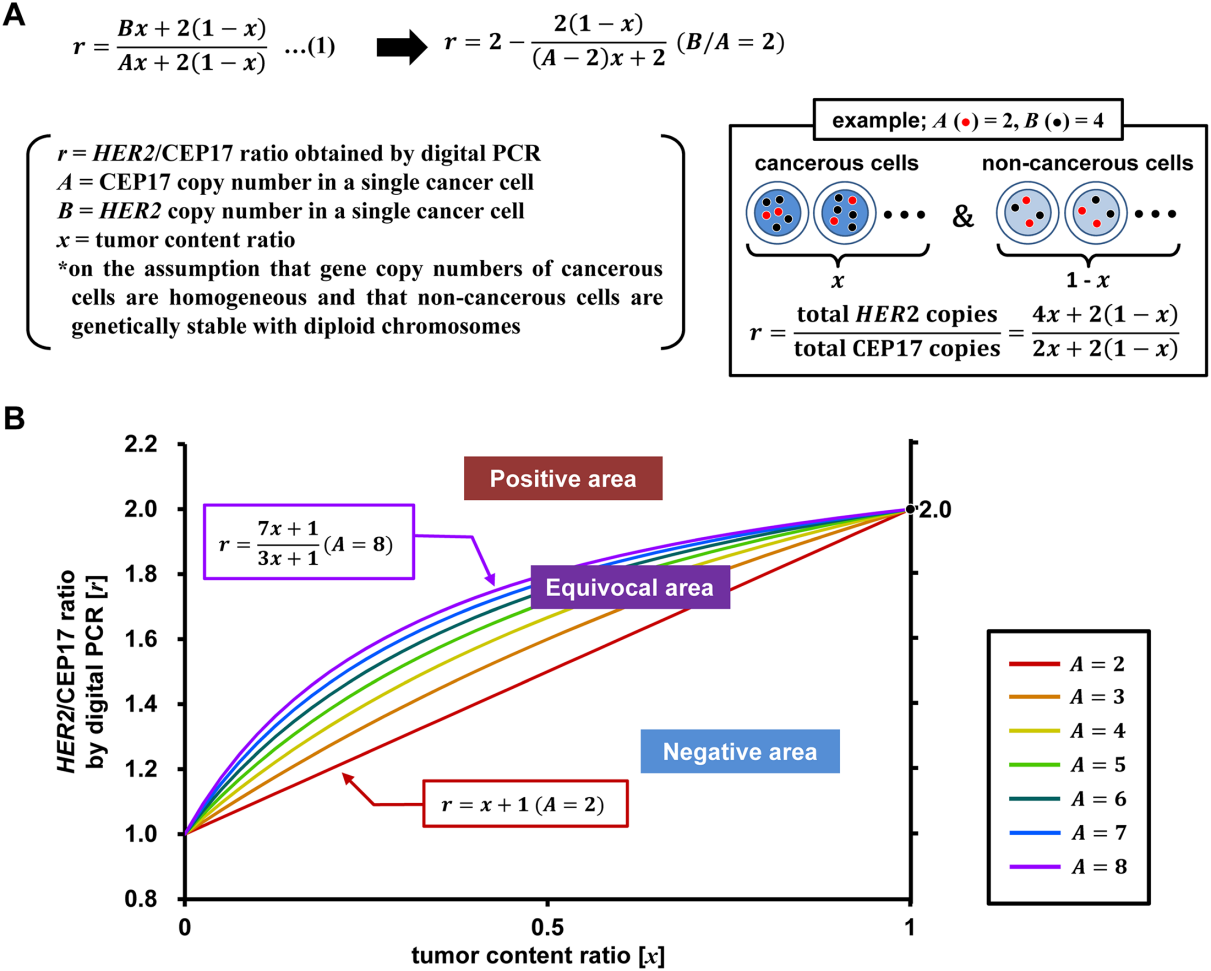
Equations and TC chart. (A) Mathematical representation of the correlation between *HER2*/CEP17 ratios obtained by digital PCR [*r*] and TCR [*x*] (upper). When [*B*/*A* = 2], [*r*] is represented by the right side of the equation (indicated by an arrow), and the right-hand member of the equation indicates a monotonic increase. A schematic model is shown for [*A* = 2] and [*B* = 4] (lower right). (B) TC chart demonstrated by Eq (1). The relationship can be plotted as a straight line (red) represented by [*r* = *x* + 1], which is the threshold between negative and equivocal areas. As the value of [*A*] exceeds 2.0, lines become increasingly convex and curve upwards according to this equation. Lines converge at the coordinates (0,1) and (1,2). In clinical samples, [*A*] was limited to a value between 2 and 8. The area enclosed by the straight red line [*r* = *x* + 1](*A* = 2) and the purple line curving upwards [*r* = (7*x* + 1)/(3*x* + 1)](*A* = 8) was designated as the equivocal area, and the area above the purple line was designated as the positive area.

The *HER2*/CEP17 ratio was determined based on three variables: TCR and absolute copy numbers of *HER2* and CEP17. Only the relative ratios of the latter two can be obtained by digital PCR using the tumor specimen without isolating single cells. The *HER2*/CEP17 ratio obtained by digital PCR [*r*] was expressed as follows, based on the assumption that gene copy numbers of cancer cells are homogeneous and that non-cancerous cells are genetically stable with diploid chromosomes (Fig 1A and 1B):

$$r=\frac{Bx+2\left(1-x\right)}{Ax+2\left(1-x\right)}$$ (1)

where [*r*] is the ratio of *HER2* to CEP17 obtained by digital PCR (0 < *r*); [*x*] is TCR (0 ≤ *x* ≤ 1); [*A*] is CEP17 copy number in a single cancer cell (0 ≤ *A*); and [*B*] is *HER2* copy number in a single cancer cell (0 ≤ *B*). Hereafter, the two-dimensional scatterplot represented by Eq (1) is referred to as the TC chart.

In the present study, [*x*] was determined from HE-stained specimens using image analysis software, such as Tissue Studio image analysis software for the biopsy specimens and Hybrid Cell Count software for the surgical specimens, respectively; and [*r*] and [*x*] were determined from measurements.

Eq (1) was represented graphically as the TC chart, with [*r*] and [*x*] as the vertical and horizontal axes, respectively (Fig 1B). The ratio of [*B*/*A*] was the reference value for determining *HER2* gene amplification. Indeed the actual copy number of CEP17 [*A*] and *HER2* [*B*] could not be determined without performing *HER2*-DISH. However, a practical evaluation of *HER2* and CEP17 copy numbers by DISH found that [*A*] ranged from 2.0 to 6.0 (between 40 and 117 CEP17 signals among 20 cancer cells in S1 and S2 Tables) and never exceeded 8.0 in clinical gastric cancer samples. According to our experience of 164 consecutive cases of gastric cancer (Ushiku T *et al*. unpublished data), CEP17 copy number is 1.0–5.7, median 2.5, and monosomy, defined as [*A* < 1.5], is quite rare in only two cases (1.2%). Thus, the range of [*A*] was set as [2 ≤ *A* ≤ 8] with a safety margin.

We defined *HER2* amplification-negative as [*B*/*A* < 2] or [*B* < 2*A*]. [*Bx* + 2(1 − *x*)] was then expressed as follows:

Bx+2(1−x)<2Ax+2(1−x) (2)

Both sides of Eq (2) were divided by [*Ax* + 2(1 − *x*)]:

$$\frac{Bx+2\left(1-x\right)}{Ax+2\left(1-x\right)}<\frac{2Ax+2\left(1-x\right)}{Ax+2\left(1-x\right)}$$ (3)

Combining with Eqs (1) and (3) yielded the following:

$$r<\frac{2Ax+2\left(1-x\right)}{Ax+2\left(1-x\right)}=2-\frac{2\left(1-x\right)}{Ax+2\left(1-x\right)}$$ (4)

$$r<2-\frac{2\left(1-x\right)}{\left(A-2\right)x+2}$$ (5)

Eq (5) was a condition equivalent to [*B*/*A* < 2]. The right side member of Eq (5) indicated a monotonic increase according to the variable [*A*](2 ≤ *A* ≤ 8); incorporation of [*A* = 2], which was the minimum value of [*A*], into Eq (5) gave the following:

r<x+1 (6)

Therefore, whenever Eq (6) was satisfied, the *HER2* amplification-negative condition [*B*/*A* < 2] was true for any value of [*A*](2 ≤ *A* ≤ 8).

On the other hand, the definition of *HER2* amplification-positive was [*B*/*A* ≥ 2] or [*B* ≥2*A*]. In this case, [*Bx* + 2(1 − *x*)] was expressed as follows:

Bx+2(1−x)≥2Ax+2(1−x) (7)

Eq (7) was processed in the same way as Eqs (2)–(5) and was expressed as follows:

$$r\geq 2-\frac{2\left(1-x\right)}{\left(A-2\right)x+2}$$ (8)

Eq (8) was a condition equivalent to [*B*/*A* ≥ 2]; the right side member of the equation indicated a monotonic increase. Incorporation of [*A* = 8], which was the maximum value of [*A*], into Eq (8) yielded the following:

$$r\geq 2+\frac{2\left(1-x\right)}{\left(8-2\right)x+2}=\frac{7x+1}{3x+1}$$ (9)

Thus, whenever Eq (9) was satisfied, the *HER2* amplification-positive condition [*B*/*A* ≥ 2] was true for any value of [*A*](2 ≤ *A* ≤ 8).

When Eqs (6) and (9) were plotted on the TC chart, the area above the line curving upwards [*r* = (7*x* + 1)/(3*x* + 1)](*A* = 8) was designated as the positive area, and the area below the straight line [*r* = *x* + 1](*A* = 2) was the negative area. The area enclosed by the lines [*r* = *x* + 1] and [*r* = (7*x* + 1)/(3*x* + 1)] was an indefinite zone depending on the value of [*A*], which was designated as the equivocal area and defined as follows (Fig 1B):

$$x+1\leq r<\frac{7x+1}{3x+1}$$ (10)

When [*x*] was 0 (containing no cancer cells), all lines converged at coordinates (0,1) based on the assumption that non-cancerous cells are genomically stable. When [*x*] was 1.0 (consisting of only cancer cells), lines passed through the coordinates (1,2) independently of values for [*A*] and [*B*].

### Comparison of digital PCR and *HER2*-DISH in cultured cells

We validated the theoretical Eq (1) and the TC chart by a stepwise mixture system of cell lines. The Eq (1) was obtained using actual measured values of [*A*] and [*B*]obtained by *HER2*-DISH in cell lines, or using the calculated [*B′*] from [*A*] and measured [*r*].

**Fig 2:**
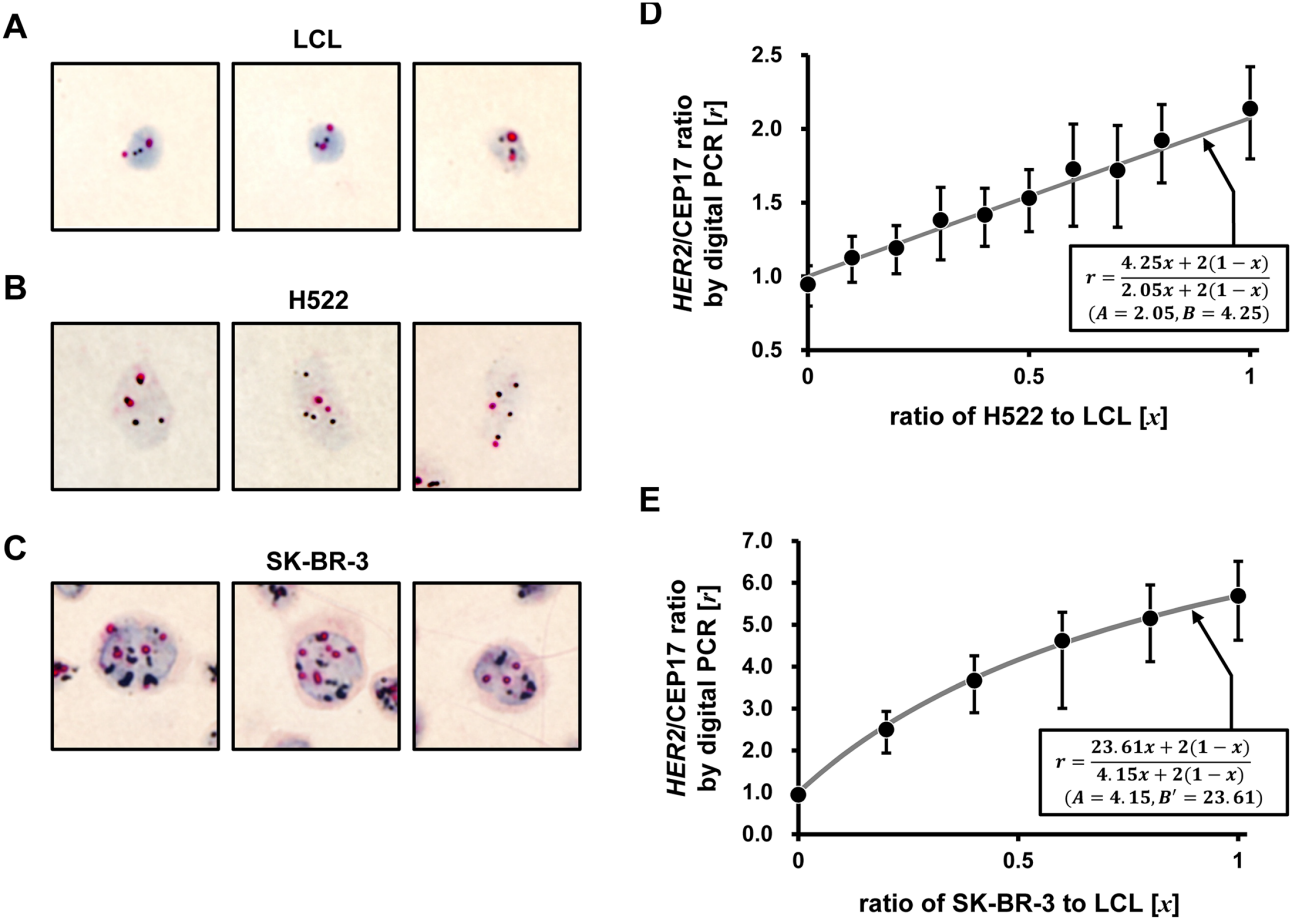
*HER2*-DISH in cell blocks and TC chart in the stepwise mixture system. (A–C) Micrographs of *HER2*-DISH of LCL, H522, and SK-BR-3 FFPE cell blocks. *HER2* signals are shown as black dots or clusters, and CEP17 signals are shown as red dots. *HER2* and CEP17 copy numbers measured by *HER2*-DISH and digital PCR are shown in Table 2. (A) The LCL was genomically stable with two pairs of signals corresponding to *HER2* and CEP17, mimicking non-cancerous stromal or inflammatory cells. (B) In H522 cells, both *HER2* and CEP17 signals appeared as dots and individual signals were discernible. (C) In SK-BR-3 cells, *HER2* signals formed clusters and exact measurements were difficult. CEP17 signals varied between 3 and 7, with an average score of 83/20 = 4.15. (D, E) Genomic DNA of H522 and SK-BR-3 cells was mixed with the LCL genome in a stepwise manner and analyzed by digital PCR. The horizontal axis represents the ratio of H522 or SK-BR-3 to LCL, corresponding to TCR [*x*] in clinical cases; the vertical axis represents the ratio of HER2 to CEP17 in digital PCR [*r*]. [*A*] and [*B*] were determined by *HER2*-DISH (Table 1). (D) In H522, [*A* = 2.05] and [*B* = 4.25], yielding [*r* = (4.25*x* + 2(1 − *x*))/(2.05*x* + 2(1 − *x*))] (solid gray line). Plotted data were approximated by the theoretical line. (E) In SK-BR-3 cells, [*A* = 4.15], but *HER2* copy number [*B*] was difficult to determine due to cluster formation. *HER2* copy number [*B′*] was predicted [*B′* = 5.69 × 4.15 = 23.61] based on digital PCR data [*r*] and CEP17 copy number [*A*]. The equation [*r* = (23.61*x* + 2(1 − *x*))/(4.15*x* + 2(1 − *x*))] is represented by the solid gray line. Plotted data were approximated by the theoretical line.

Relative copy numbers of *HER2* and CEP17 (*HER2*/CEP17) in LCL and H522 and SK-BR-3cells were evaluated by *HER2*-DISH using FFPE cell blocks and by digital PCR (Table 2). *HER2* signals appeared as discrete dots in nuclei by *HER2*-DISH (Fig 2A and 2B); *HER2* copy number in individual cells was nearly two in LCL and four in H522 cells. In contrast, SK-BR-3 cells showed clustered *HER2* signals that were difficult to distinguish individually (Fig 2C); their numbers were arbitrarily set to the maximum values of 7, 14, and 21 according to cluster sizes. Thus, a lower *HER2*/CEP17 ratio was obtained by *HER2*-DISH than by digital PCR.

**Table 2:** *HER2*/CEP17 ratios from *HER2*-DISH and digital PCR in H522 and SK-BR-3 cells and LCL.

	*HER2*-DISH (20 cells)			digital PCR		
	*HER2* count	CEP17 count	*HER2*/CEP17 ratio	*HER2* count	CEP17 count	*HER2*/CEP17 ratio
**LCL**	40	40	**1.00**	383	398	**0.95**
**H522**	85	41	**2.07**	513	310	**2.14**
SK-BR-3*	381	83	**4.59**	639	206	**5.69**

*SK-BR-3 showed significant discrepancy in counts between *HER2*-DISH and digital PCR (*p* < 0.01).

H522 and SK-BR-3 cell genomic DNA was mixed with that of the LCL in a stepwise manner and the mixtures were analyzed by digital PCR. In H522 cells, *HER2* [*B*] and CEP17 [*A*] copy numbers were calculated from *HER2*-DISH as follows: [*A* = 41/20 = 2.05] and [*B* = 85/20 = 4.25]. When the values of [*A*] and [*B*] were applied to Eq (1), the data could be described by the reference line [*r* = (4.25*x* + 2(1 − *x*))/(2.05*x* + 2(1 − *x*))] (Fig 2D). In the case of SK-BR-3 cells, CEP17 copy number [*A*] was [*A* = 83/20 = 4.15]; however, as described above, *HER2* signals were clustered, which decreased the estimated copy number. Therefore, applying [*A* = 4.15], [*r* = 5.69], and [*x* = 1.0] to Eq (1), the estimated *HER2* copy number [*B′*] was presumed to be [*B′* = 5.69×4.15 = 23.61]. Hence, [*A* = 4.15] and [*B′* = 23.61] whilethe reference line was [*r* = (23.61*x* + 2(1 − *x*))/(4.15*x* + 2(1 − *x*))] (Fig 2E). Plotted digital PCR data from SK-BR-3 cells mixed with the LCL conformed to this line rather than [*r* = (19.50*x* + 2(1 − *x*))/(4.15*x* + 2(1 − *x*))], which was based on *HER2*-DISH data for which [*B* = 19.50]. These results demonstrate the validity of the TC chart—i.e., that it has sufficient quantitativity of digital PCR to distinguish HER2-amplified from non-amplified cells.

### Concordance between TC chart for digital PCR and HER2-IHC/-DISH in clinical biopsy samples

**Fig 3:**
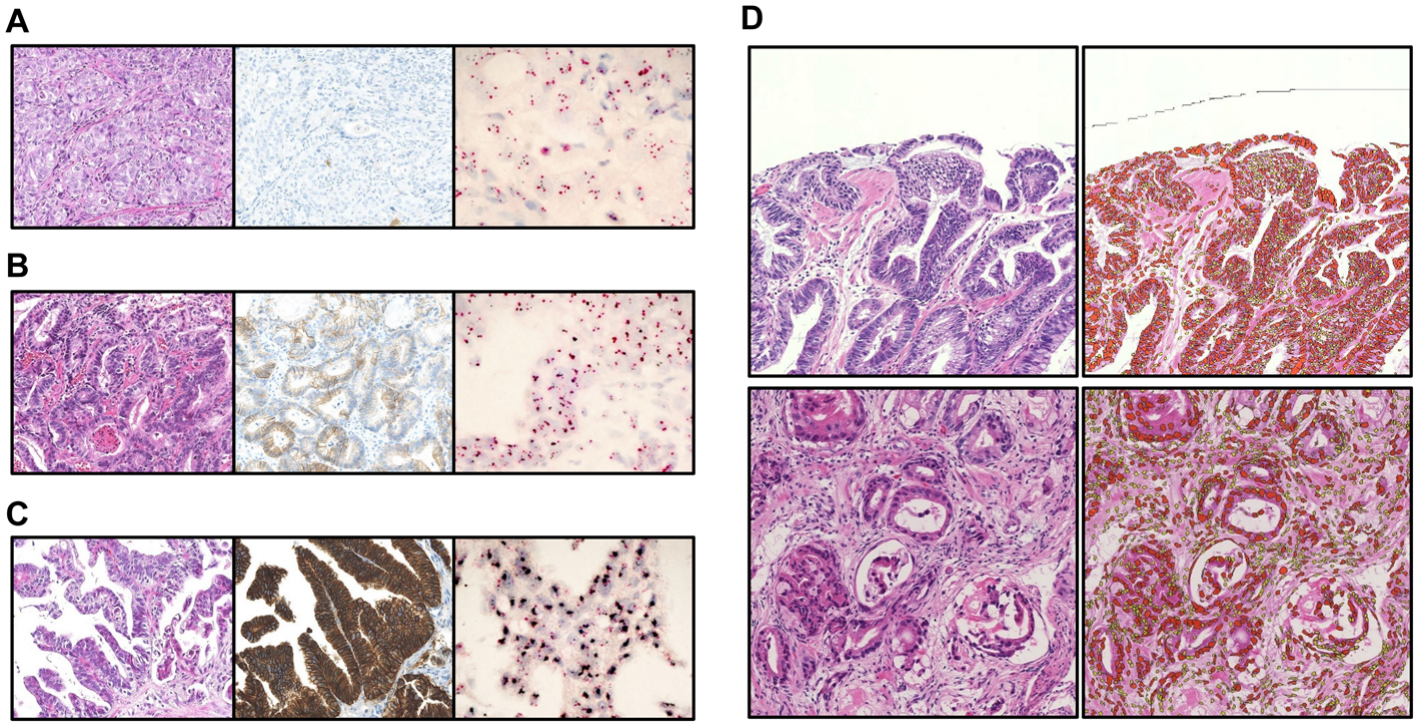
Histological analysis of clinical gastric cancer biopsy specimens and estimation of TCR using Tissue Studio. (A–C) Representative cases are shown with HE staining, HER2-IHC, and *HER2*-DISH. (A) Case #37: HER2-IHC score = 0, *HER2*-DISH ratio = 1.09. (B) Case #6: HER2-IHC score = 2+, *HER2*-DISH ratio = 2.83. (C) Case #25: HER2-IHC score = 3+, *HER2*-DISH ratio = 6.56. (D) TC rate was calculated based on the ratio of nuclei counts of whole nucleated and cancerous cells, which were semi-automatically distinguished by nucleus size—with cancerous cells showing enlarged nuclei—by means of Tissue Studio image analysis software.

Gastric cancer biopsy specimens (n = 44) from 32 patients were analyzed by digital PCR. The *HER2* status of each specimen was determined by HER2-IHC and -DISH (Fig 3A–3C). The estimated values were consistent with manual counts made by a pathologist (KM). TC rate was determined from images by distinguishing nuclei of cancerous from those of non-cancerous cells (Fig 3D).

**Fig 4:**
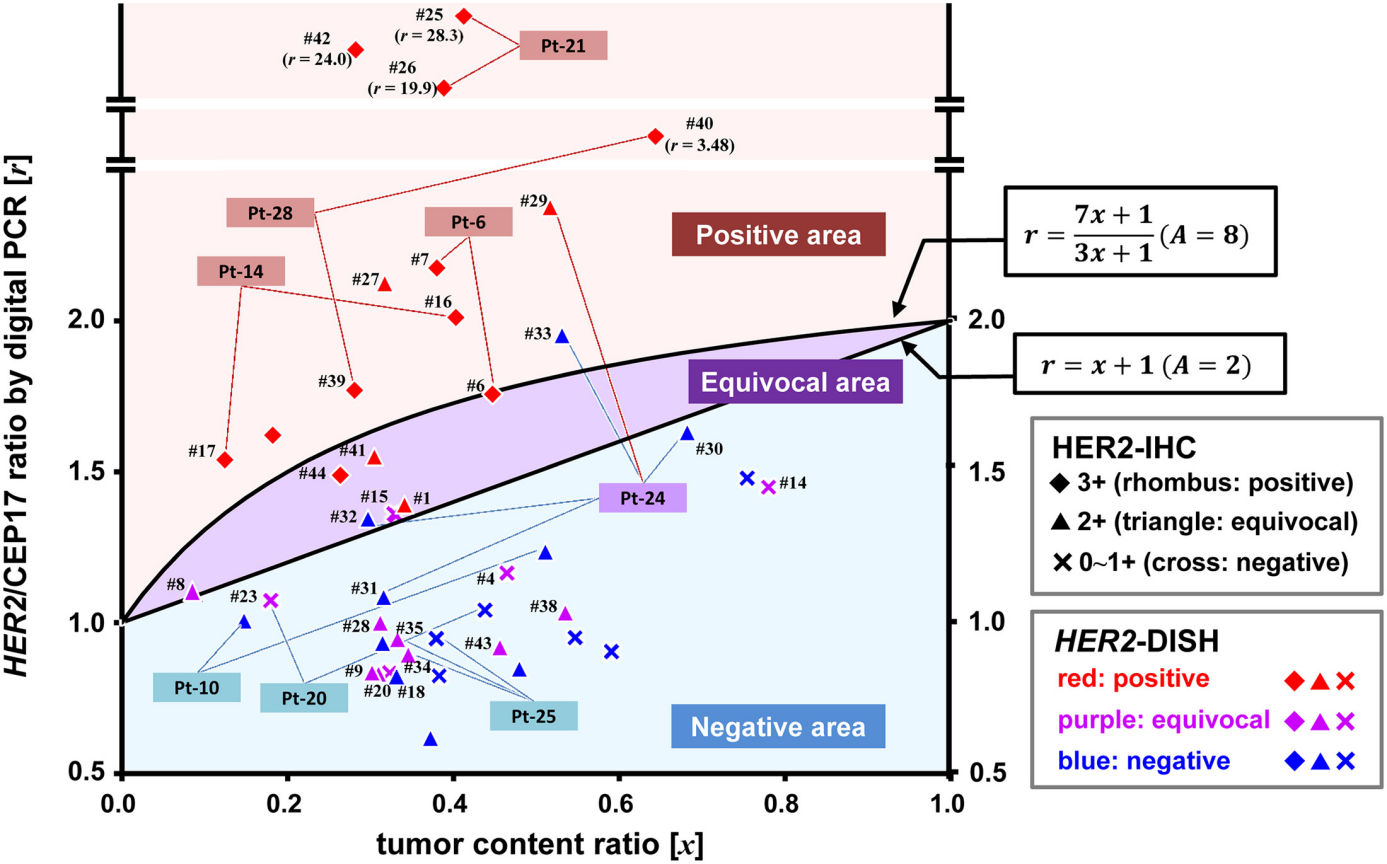
TC chart-assisted digital PCR analysis of clinical gastric cancer biopsy specimens. Each case was evaluated by HER2-IHC and digital PCR, and then validated by *HER2*-DISH (Table 3 and S1 Table). The results are plotted on the TC-chart. The findings of HER2-IHC are represented by symbols as follows; 3+ (positive) as rhombi; 2+ (equivocal) as triangles; 0~1+ (negative) as cross-mark. The results of *HER2*-DISH are depicted by colors as follows; positive, red; equivocal, purple; negative, blue. Multiple cases taken from the same patients (Pt) are highlighted. The vertical axis between 0.5 and 2.5 was linearly ordered and the upper area was logarithmically ordered. There were no positive cases (red rhombi) in the negative area, demonstrating that digital PCR can screen out negative cases with high specificity. On the other hand, cases plotted in the equivocal area were not always positive, as determined by CEP17 copy number. Three cases (#25, #26, and #42) showed markedly high scores and a clustering pattern in *HER2*-DISH (Fig 3C).

Digital PCR data and TCR of each case were plotted on the TC chart (Fig 4), including data for *HER2*-DISH-positive (red), -equivocal (purple) and -negative (blue), as well as HER2-IHC-positive (rhombi), -equivocal (triangles), and -negative (cross-marks) samples. Clinicopathological information on representative and total cases is shown in Table 3 and S1 Table respectively. There were no *HER2*-DISH-positive cases below the line[*r* = *x* + 1]; however, one *HER2*-DISH-negative (#32), two equivocal (#8 and #15), and three positive (#1, #41, and #44) cases were observed in the equivocal area. In addition, there was one *HER2*-DISH-negative case in the positive area (#33). Three cases (#25, #26, and #42) showed markedly high digital PCR values (28.25, 19.99, and 24.00, respectively). In *HER2*-DISH, these cases showed clustered *HER2* signals (Fig 3C) that were difficult to quantify, as in SK-BR-3 cells. Eq (1) was also expressed as follows:

$$\frac{B}{A}=r+\;\frac{2\left(r-1\right)\left(1-x\right)}{Ax}$$ (11)

**Table 3:** Information on HER2 status and clinicopathological factors for representative cases.

Pt	#	Sex	Hist	TCR [*x*]	dPCR	TC chart area	HER2-IHC	*HER2*-DISH (20 cells)			*B*/*A*	*B’*
					ratio [*r*]			*HER2* count	CEP17 count	*HER2*/CEP17		
1	#1	M	I	0.341	1.39	Equivocal	2+	261	57	**4.58**	**1.92**	3.96
32	#44	M	D	0.264	1.49	Equivocal	3+	158	60	**2.63**	**2.40**	4.47
7	#8	M	D	0.087	1.10	Equivocal	2+	106	79	**1.34**	**1.63**	4.35
13	#15	M	I	0.330	1.36	Equivocal	1+	102	55	**1.86**	**1.89**	3.74
24	#32	M	D	0.297	1.35	Equivocal	2+	64	52	**1.23**	**1.99**	3.51
29	#41	M	D	0.305	1.55	Equivocal	2+	132*	87	1.52*	**2.13**	6.75
24	#33	M	D	0.531	1.95	Positive	2+	65	62	**1.05**	**2.49**	6.05
24	#29	M	I	0.517	2.38*	Positive	2+	111	48	**2.31**	**3.45**	5.71
21	#25	M	I	0.414	28.25	Positive	3+	420	64	**6.56**	**52.36**	90.4
21	#26	M	I	0.390	19.99	Positive	3+	420	63	**6.67**	**38.85**	62.97
30	#42	M	D	0.283	24.00	Positive	3+	420	59	**7.12**	**63.51**	70.80

Pt, patient number; #, case number; Hist, Histology; TCR, tumor content ratio; dPCR, digital PCR; M, male; I, intestinal type; D, diffuse type.

*132/20 = 6.6 HER2 signals/cell (> 6.0) despite *HER2/*CEP17 ratio = 1.52 (DISH < 2.0).

Theoretically estimated [*B*/*A*] was calculated by application of measured [*r*] and [*x*] and measured [*A*], which was determined from counts of CEP17 signals obtained by *HER2*-DISH in 20 cancer cells (Table 3 and S1 Table). [*B′*]was also obtained by measuring [*r*] and [*A*] and [*x* = 1], as described for SK-BR-3 cells.

Multiple cases were evaluated for eight patients (Fig 4 and S1 Table). All cases in each patient showed the same results for DISH and digital PCR except for Patient-24 (Pt-24); one of five cases (#29) was positive and two (#30 and #31) were negative for both measurements. Two cases negative for DISH were allocated to positive (#33) and equivocal (#32) areas in the TC chart (Table 3 and S1 Table).

In total, 22 HER2-IHC cases had a score of 2+ (Table 4), comprising three positive, 15 negative, and four equivocal cases based on the TC chart of digital PCR. Positive and negative results were completely concordant with those of *HER2*-DISH. Of the four equivocal cases with IHC score 2+, one (#1) was positive and the others (#8, #32, and #41) were negative, as determined by *HER2*-DISH. The estimated [*B*/*A*] value was accordingly ≥ 2.0 in case #1 and < 2.0 in the latter three, except for case #41, the estimated [*B*/*A*] of which was 2.12.

**Table 4:** Summary of correlations between immunohistochemistry and TC chart-assisted digital PCR data.

	TC chart			
**HER2-IHC**	**Symbol**	**Positive area**	**Negative area**	**Equivocal area**
**0/1+**	**×**	0	10	1*^1^
**2+**	▲	3*^2^	15	4*^3^
**3+**	◆	10	0	1*^4^
***HER2*-DISH**	**Symbol color**	**Positive area**	**Negative area**	**Equivocal area**
**Negative**	Blue	1	14	1
**Equivocal**	Purple	0	11	2
**Positive**	Red	11	0	4

*^1^: DISH-equivocal (#15).

*^2^: two samples were DISH-positive (#27 and 29) and one sample was DISH-negative (#33).

*^3^: two was DISH-positive (#1 and 41), one was DISH-equivocal (#15), and the other was DISH-negative (#32).

*^4^: DISH-positive (#44).

### Evaluation of intratumoral HER2 heterogeneity in surgical specimens

**Fig 5:**
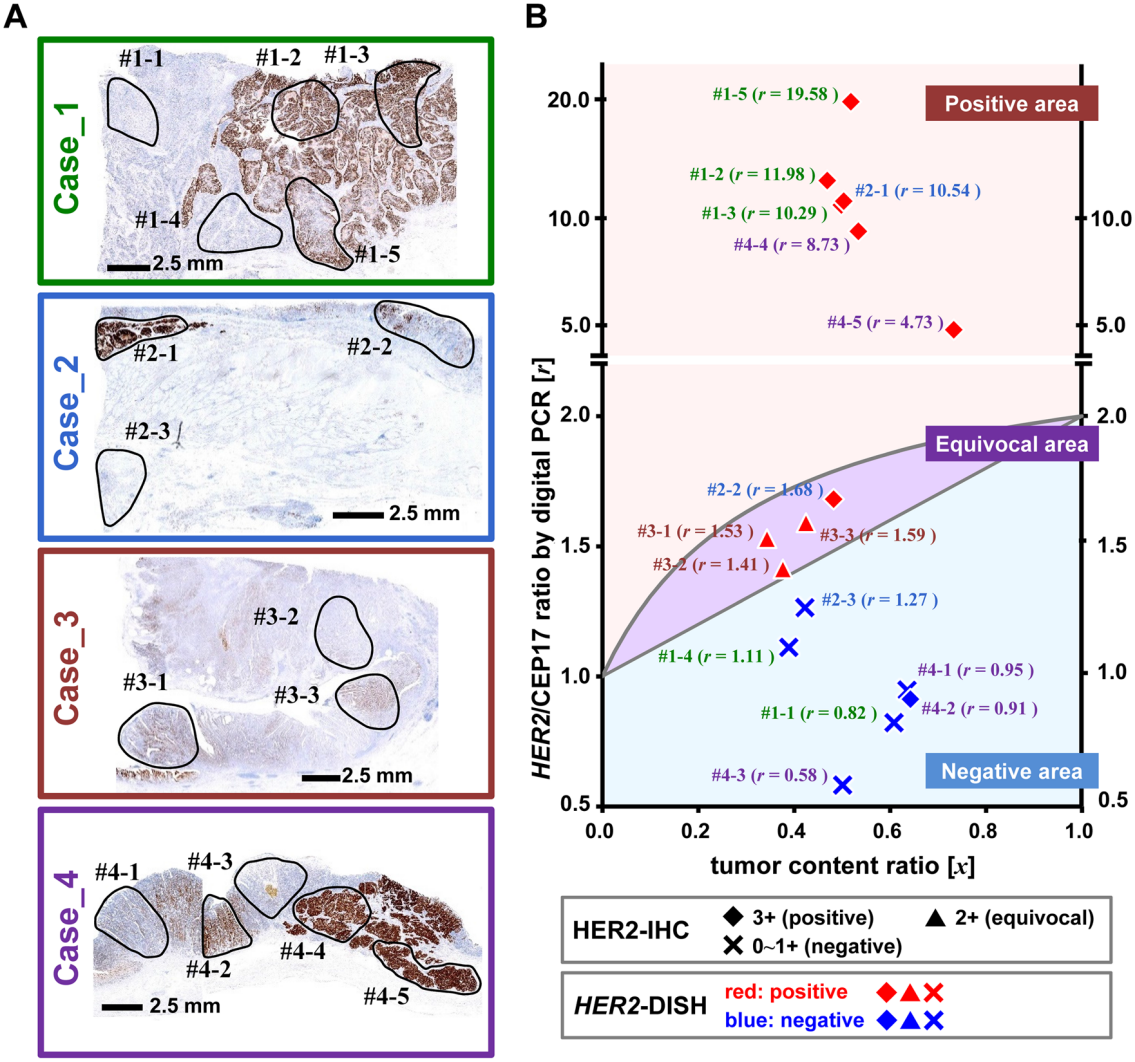
Evaluation of intratumoral HER2 heterogeneity in surgical specimens by TC chart-assisted digital PCR analysis. (A) Four surgical resected cases were evaluated in each representative section by a combination of HE staining, HER2-IHC, and -DISH. Each case is indicated by colors; Case_1, dark green; Case_2, marine blue; Case_3, dark red; Case_4, dark purple. High magnification views of HE staining, HER2-IHC, and *HER2*-DISH are shown in S1 Fig. Regions selected for DNA extraction are demarcated by a solid black line. Case_1, _2, and _4 show heterogeneity in HER2 status. HER2-overexpression areas in HER2-IHC generally corresponded to *HER2* amplification areas, with clustered or multiple *HER2* signals in *HER2*-DISH. Region #2–2 includes components showing both HER2-IHC 3+ and 0–1. Case_3 consists of relatively homogenous components showing a HER2-IHC 2+ (equivocal) pattern. (B) The digital PCR results are plotted on the TC-chart in the same manner as in Fig 4. Each plot is marked with the sample name and [*r*] value in the color specific to each case. The vertical axis between 0.5 and 2.2 was linearly ordered and the upper area was logarithmically ordered.

Intratumoral HER2 heterogeneity was evaluated by a combination of HER2-IHC/-DISH and digital PCR (Fig 5, S1 Fig and S2 Table). Case_1, 2, and 4 showed distinct intratumoral heterogeneity consisting of HER2-IHC-positive and -negative lesions in a cluster, while Case_3 generally showed an equivocal HER2-IHC expression pattern. Importantly, HER2-positive lesions were accessible from the lumen of the stomach in Case_1 (#1–2, #1–3), Case_2 (#2–1, #2–2), and Case_4 (#4–4).

All HER2-IHC/-DISH-positive cases were plotted in the positive area. Three samples of Case_3 (#3–1, -2, and -3) showing HER2-IHC (equivocal) and *HER2*-DISH (positive) were plotted in the equivocal area. Sample #2–2 was plotted in the equivocal area because region #2–2 covered a relatively large area containing both HER2-positive and -negative components. Sample #4–2 showed discrepancies between HER2-IHC (positive) and *HER2*-DISH (negative) (S2 Table), with the digital PCR results corresponding to *HER2*-DISH.

## Discussion

The study is the first to attempt a categorization of gastric cancers into *HER2* amplification-negative, -equivocal, and -positive cases by using a two-dimensional scatter plot or TC chart, based on measured values of *HER2*/CEP17 ratio obtained by digital PCR [*r*] and TCR [*x*] without conventional *HER2*-DISH or HER2-IHC information. Thus, an analysis by TC chart is independent of CEP17 and *HER2* copy numbers ([*A*] and [*B*]) in a single cancer cell, which can normally be obtained by *HER2*-DISH.

The digital PCR method has been applied to the evaluation of copy number of virus [28], fetal chromosomal aneuploidy [29], and oncogenes in various cancer tissues [30, 31]. A major problem that must be overcome is the correction of raw data according to the tumor content of specimens; gastric cancer tissues generally contain larger amounts of non-cancerous cell infiltration, e.g. interstitial or inflammatory cells. In the present study, we used image analysis to obtain a semi-automated estimation of tumor cell content and developed the TC chart to classify data into three categories—i.e., amplified, non-amplified, and equivocal. It might be possible to purify cancer cells from FFPE tissue sections by microdissection, but this is too laborious for routine clinical practice, even if it is more precise in theory. Using this approach for biopsy specimens, the number of HER2-IHC-equivocal cases (2+) was reduced from 22 to six, thereby the need for confirmation by *HER2*-DISH could be obviated to less than 30% of cases. The frequency of *HER2* amplification varies across gastric cancer histological types, from 20%–30% in the intestinal to < 10% in the diffuse type [32]. Therefore, an effective screening method is required in routine clinical practice, especially in countries with a high prevalence of gastric cancer. TC chart can be also used with other PCR-based method, such as quantitative PCR for *HER2* amplification [33].

There are additional advantages to the digital PCR protocol. Firstly, it is simple relative to ISH, which requires skill and is labor-intensive, comprising multiple steps. Secondly, fewer cases are required for validation than are needed for *HER2*-DISH, making it cost-effective. Finally, it is only necessary to design specific primers that can be applied to genes other than *HER2*, making it a more versatile technique than ISH or IHC.

A major problem with current HER2-IHC and -ISH protocols is the fact that HER2-IHC 0/1+ cases can show positive signals by *HER2*-ISH [19]. This was not observed in the present study, but two HER2-IHC 1+ samples (#14 and #15) had values of 1.98 and 1.89 by *HER2*-DISH. The average HER2 copy number in both samples were 4.15 and 5.05 by *HER2* single probe assay, respectively (S1 Table). The update ASCO/CAP guideline for breast cancer has adopted the criteria of equivocal for *HER2* amplification, that is, [*HER*2/CEP17 ratio < 2.0] and absolute value of [average *HER*2 copy number ≥ 4.0 and < 6.0 signals/cell] by *HER2*-DISH [19]. If this applied to both cases (#14 and #15), these were *HER2*-DISH-equivocal. Moreover, there was one case (#41) showing negative *HER2*/CEP17 ratio and positive average *HER2* signals in a cancer cell (Table 3). Further studies are necessary in gastric cancer to prove whether single probe analysis is sufficient or not.

Aneusomy of chromosome 17 (monosomy and polysomy) may affect *HER2* amplification measurements by the *HER2*/CEP17 ratio, although its clinical importance is unknown in the absence of HER2 overexpression on IHC [19]. In this study, there was no case showing average CEP17 copy number [*A*] less than 2.0 (S1 and S2 Tables). Polysomy may be more common in gastric cancer, but the average CEP17 copy number [*A*] did not exceed 6.0. Since selection bias does not affect chromosomal regions without oncogenes, CEP17 copy number may be relatively stable and easy to count. Jacquemier *et al*. reported well concordance between FISH and the quantitative real time PCR (qPCR) as alternative techniques in a large series of breast cancer patients [33]. They pointed out that the reliability of qPCR was increased by preparing multiple primer sets for chromosomal regions. It is thus possible to adopt another set of primers for CEP17 to determine average values of [*A*] by digital PCR.

The histological heterogeneity of tumors is a major problem associated with *HER2* amplification measurements, especially in gastric cancer. This was illustrated in the present study by Patient-24 (cases #29–#33) of biopsy examination. Amplification was demonstrated by DISH in only one of five samples that showed the intestinal type histology. On the other hand, the TC chart-assisted digital PCR analysis classified two samples as amplified, one as equivocal, and only two as negative. As such, this method may be able to reduce the number of samples required for measurements. Several studies have suggested that patients showing a higher copy number of *HER2* respond better to anti-HER2 monoclonal antibody treatment. Therefore, a quantitative method for further stratifying patients with advanced gastric cancer would be useful.

Intratumoral heterogeneity was also examined in surgically resected gastric cancer in the present study although the number was small, which confirmed the reliability of TC chart-assisted digital PCR analysis. Sample #4–2 was plotted to the negative area, even if it showed positivity with HER2-IHC. The *HER2*-DISH result was negative and polysomy was therefore excluded. Such a case was exceptional in gastric cancer based on results from biopsy samples. It is of worth noting that HER2-positive components appeared as a cluster, and were accessible from the lumen of the stomach. These facts illustrate that biopsy specimens may represent the tissue distribution of HER2 status when specimens are obtained from several points distant from each other within each cancer. Ye *et al*. recently reported that the risk of false negative results was 0.23% when three biopsy specimens were collected [34]. To evaluate this issue more precisely, multiple small pieces of surgical specimens should be compared, for instance by automated PCR-based analysis. Additional studies are necessary to establish a biopsy protocol for determining the status of specific molecules following targeted therapy of gastric cancer.

## Supporting Information

S1 Fig High magnification views of surgical specimens. HE staining, HER2-IHC, and -DISH in surgical specimens of Case_1 to 4 in Fig 5A. Note the HER2-IHC score and *HER2*/CEP17 ratio by *HER2*-DISH in the upper left corner of each figure.
(TIF)

S1 Table Information on HER2 status and clinicopathological factors for all biopsy specimens. Red, purple, and blue colors indicate positive, equivocal, and negative HER2 status in the respective methods. Theoretically [*B*/*A*] was calculated by application of measured [*r*] and [*x*] and expected [*A*] into Eq (11) (estimated [*B* /*A*]).
(PDF)

S2 Table Information on HER2 status for all surgical specimens. Red, purple, and blue colors indicate positive, equivocal, and negative of HER2 status in the respective methods.
(PDF)
