# Supplementary figures and images for: Tumor Content Chart-Assisted HER2/CEP17 Digital PCR Analysis of Gastric Cancer Biopsy Specimens
Source: PLoS One. 2016 Apr 27;11(4):e0154430. doi: 10.1371/journal.pone.0154430 (PMC4847903; doi:10.1371/journal.pone.0154430)

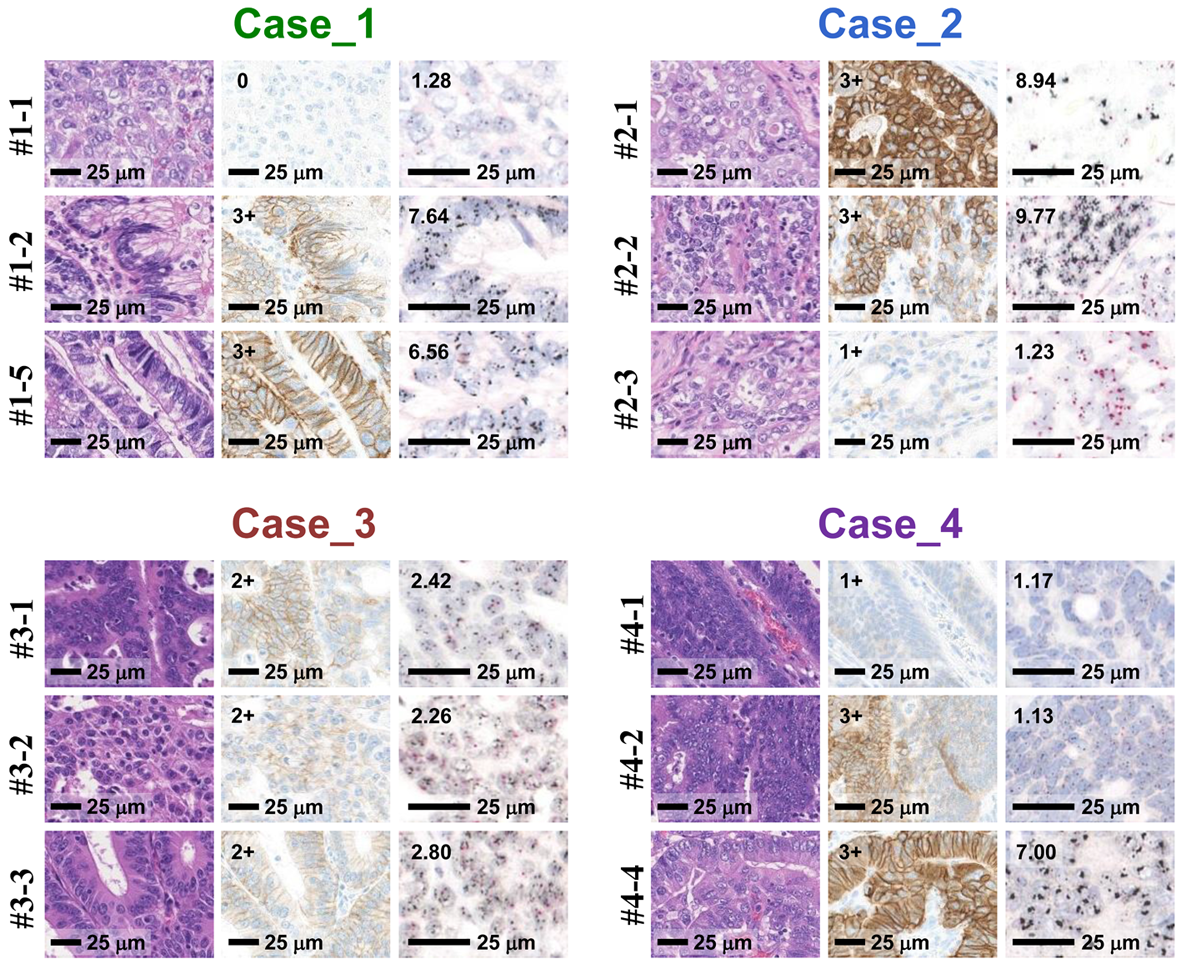

Supplement: S1 Fig — HE staining, HER2-IHC, and -DISH in surgical specimens of Case_1 to 4 in Fig 5A. Note the HER2-IHC score and HER2/CEP17 ratio by HER2-DISH in the upper left corner of each figure. (TIF) [file pone.0154430.s001.tif]
