# Supplementary material for: Tumor Content Chart-Assisted HER2/CEP17 Digital PCR Analysis of Gastric Cancer Biopsy Specimens
Source: PLoS One. 2016 Apr 27;11(4):e0154430. doi: 10.1371/journal.pone.0154430 (PMC4847903; doi:10.1371/journal.pone.0154430)
Supplement: S1 Table — Red, purple, and blue colors indicate positive, equivocal, and negative HER2 status in the respective methods. Theoretically [B/A] was calculated by application of measured [r] and [x] and expected [A] into Eq (11) (estimated [B /A]). (PDF) [file pone.0154430.s002.pdf]

S1 table. **Information on HER2 status and clinicopathological factors for all biopsy specimens.**

Pt	#	Age	Sex	Hist	TCR [x]	digital PCR			TC chart area	HER2 -IHC	HER2-DISH (20 cells)			B/A
						HER2 count	CEP17 count	ratio [*r*]			HER2 count	CEP17 count	*HER2*/ CEP17	
1	#1	80	M	I	**0.341**	117	86	**1.39**	**Equivocal**	**2+**	**261**	57	**4.58**	1.92
2	#2	67	M	D	**0.182**	270	180	**1.62**	**Positive**	**3+**	**193**	47	**4.11**	3.99
3	#3	82	F	I	**0.754**	348	257	**1.48**	**Negative**	**1+**	**62**	45	**1.38**	1.62
4	#4	85	F	I	**0.464**	578	536	**1.17**	**Negative**	**1+**	**107**	93	**1.15**	1.25
5	#5	63	M	D	**0.546**	427	441	**0.95**	**Negative**	**0**	**46**	46	**1.00**	0.91
6	#6	67	F	I	**0.447**	691	559	**1.76**	**Positive**	**3+**	**164**	58	**2.83**	2.41
	#7			I	**0.380**	444	251	**2.18**	**Positive**	**3+**	**306**	82	**3.73**	3.12
7	#8	74	M	D	**0.087**	276	256	**1.10**	**Negative**	**2+**	**106**	79	**1.34**	1.63
8	#9	63	M	D	**0.302**	536	585	**0.84**	**Equivocal**	**2+**	**99**	117	**0.85**	0.71
9	#10	79	M	I	**0.372**	337	466	**0.62**	**Negative**	**2+**	**69**	105	**0.66**	0.38
10	#11	79	F	I	**0.511**	315	267	**1.24**	**Negative**	**2+**	**45**	60	**0.75**	1.39
	#12			I	**0.147**	441	439	**1.01**	**Negative**	**2+**	**46**	57	**0.81**	1.05
11	#13	75	F	I	**0.382**	494	548	**0.82**	**Negative**	**0**	**66**	93	**0.71**	0.69
12	#14	27	F	D	**0.780**	127	90	**1.45**	**Negative**	**1+**	**83**	42	**1.98**	1.57
13	#15	78	M	I	**0.330**	307	241	**1.36**	**Equivocal**	**1+**	**102**	55	**1.86**	1.89
14	#16	66	M	D	**0.403**	727	593	**2.01**	**Positive**	**3+**	**188**	45	**4.18**	3.34
	#17			D	**0.124**	442	328	**1.54**	**Positive**	**3+**	**212**	62	**3.42**	4.00
15	#18	63	M	D	**0.332**	320	370	**0.82**	**Negative**	**0**	**108**	96	**1.13**	0.67
16	#19	72	M	I	**0.479**	291	330	**0.85**	**Negative**	**2+**	**71**	81	**0.88**	0.77
17	#20	63	M	I	**0.309**	261	302	**0.83**	**Negative**	**2+**	**119**	114	**1.04**	0.70
18	#21	69	M	I	**0.314**	459	478	**0.93**	**Negative**	**2+**	**67**	54	**1.24**	0.82
19	#22	76	F	D	**0.331**	97	116	**0.82**	**Negative**	**2+**	**56**	70	**0.80**	0.61
20	#23	69	F	D	**0.179**	76	71	**1.07**	**Negative**	**1+**	**97**	56	**1.73**	1.30
	#24			D	**0.437**	105	101	**1.04**	**Negative**	**1+**	**79**	58	**1.36**	1.08
21	#25	66	M	I	**0.414**	747	90	**28.25**	**Positive**	**3+**	**420**	64	**6.56**	52.36
	#26			I	**0.390**	764	166	**19.99**	**Positive**	**3+**	**420**	63	**6.67**	38.85
22	#27	68	F	D	**0.317**	224	115	**2.13**	**Positive**	**2+**	**165**	76	**2.17**	3.41
23	#28	70	M	I	**0.312**	93	93	**1.00**	**Negative**	**2+**	**87**	64	**1.36**	1.00
24	#29	79	M	I	**0.517**	197	90	**2.38**	**Positive**	**2+**	**111**	48	**2.31**	3.45
	#30			D	**0.682**	285	190	**1.63**	**Negative**	**2+**	**61**	59	**1.03**	1.83
	#31			D	**0.316**	109	101	**1.09**	**Negative**	**2+**	**63**	59	**1.07**	1.22
	#32			D	**0.297**	125	95	**1.35**	**Equivocal**	**2+**	**64**	52	**1.23**	1.99
	#33			D	**0.531**	300	172	**1.95**	**Negative**	**2+**	**65**	62	**1.05**	2.49
25	#34	71	M	D	**0.345**	307	334	**0.89**	**Negative**	**2+**	**93**	70	**1.33**	0.77
	#35			I	**0.332**	232	243	**0.95**	**Negative**	**2+**	**105**	71	**1.48**	0.89
	#36			D	**0.379**	166	174	**0.95**	**Negative**	**1+**	**69**	63	**1.10**	0.90
26	#37	64	F	D	**0.590**	82	90	**0.91**	**Negative**	**1+**	**74**	68	**1.09**	0.87
27	#38	75	M	I	**0.535**	268	261	**1.03**	**Negative**	**2+**	**118**	87	**1.36**	1.04
28	#39	82	M	D	**0.281**	728	621	**1.77**	**Positive**	**3+**	**183**	61	**3.00**	3.06

	#40			D	**0.643**	447	170	**3.48**	**Positive**	3+	**159**	42	**3.79**	4.79
29	#41	60	M	D	**0.305**	158	106	**1.55**	**Equivocal**	2+	**132**	87	**1.52**	2.13
30	#42	81	M	D	**0.283**	753	113	**24.00**	**Positive**	3+	**420**	59	**7.12**	63.51
31	#43	72	M	I	**0.456**	125	135	**0.92**	**Negative**	2+	**100**	70	**1.43**	0.87
32	#44	82	M	D	**0.264**	278	200	**1.49**	**Equivocal**	3+	**158**	60	**2.63**	2.40

Pt, patient number; #, case number; Hist, Histology; TCR, tumor content ratio; M, male; F, female; I, intestinal type; D, diffuse type.
