# Supplementary material for: Tumor Content Chart-Assisted HER2/CEP17 Digital PCR Analysis of Gastric Cancer Biopsy Specimens
Source: PLoS One. 2016 Apr 27;11(4):e0154430. doi: 10.1371/journal.pone.0154430 (PMC4847903; doi:10.1371/journal.pone.0154430)
Supplement: S2 Table — Red, purple, and blue colors indicate positive, equivocal, and negative of HER2 status in the respective methods. (PDF) [file pone.0154430.s003.pdf]

S2 table. **Information on HER2 status for all surgical specimens.**

Pt	#	TCR [x]	digital PCR			TC chart area	HER2 -IHC	HER2-DISH (20 cells)			B/A
			HER2 count	CEP17 count	ratio [*r*]			HER2 count	CEP17 count	*HER2*/ CEP17	
1	#1-1	**0.607**	603	649	**0.82**	Negative	0	46	42	1.10	0.71
	#1-2	**0.441**	758	248	**11.98**	Positive	3+	420	55	7.64	22.12
	#1-3	**0.468**	693	157	**10.29**	Positive	3+	420	59	7.12	17.44
	#1-4	**0.388**	654	630	**1.11**	Negative	0	77	60	1.28	1.23
	#1-5	**0.487**	764	220	**19.58**	Positive	3+	420	64	6.56	31.83
2	#2-1	**0.473**	451	62	**10.54**	Positive	3+	420	47	8.94	19.58
	#2-2	**0.482**	498	356	**1.68**	Equivocal	3+	420	43	9.77	2.36
	#2-3	**0.422**	491	425	**1.27**	Negative	1+	49	40	1.23	1.63
3	#3-1	**0.343**	666	564	**1.53**	Equivocal	2+	104	43	2.42	2.47
	#3-2	**0.376**	729	677	**1.41**	Equivocal	2+	122	54	2.26	1.92
	#3-3	**0.424**	683	577	**1.59**	Equivocal	2+	129	46	2.80	2.29
4	#4-1	**0.635**	198	207	**0.95**	Negative	1+	48	41	1.17	0.92
	#4-2	**0.642**	365	389	**0.91**	Negative	3+	45	40	1.13	0.86
	#4-3	**0.500**	445	593	**0.58**	Negative	0	52	53	0.98	0.27
	#4-4	**0.501**	747	267	**8.73**	Positive	3+	420	60	7.00	13.86
	#4-5	**0.688**	733	374	**4.73**	Positive	3+	420	68	6.18	5.72

Pt, patient number; #, case number; TCR, tumor content ratio.
